# Supplementary material for: Genetic enhancers of partial PLK1 inhibition reveal hypersensitivity to kinetochore perturbations
Source: PLoS Genet. 2023 Aug 28;19(8):e1010903. doi: 10.1371/journal.pgen.1010903 (PMC10491399; doi:10.1371/journal.pgen.1010903)
Supplement: S1 Fig — NALM-6 cells were infected with two different sgRNA constructs per gene and with 2 control sgRNAs. After selection with antibiotics, cells were treated with the indicated drugs at the IC30 concentrations or with DMSO for 96 hours. Percentages of cell proliferation in the presence of the drug relative to the DMSO control for each cell line are shown. Error bars: range of values from 2 independent experiments. A. BI2536 8.1 nM. B. BI6727 12.5 nM.C. GSK461364A 8.4 nM. D. All results in parallel, showing correlation. Dashed lines: % proliferation of the 2 controls. Coordinate values used to generate graphs are available in S8 Data. (PDF) [file pgen.1010903.s001.pdf]

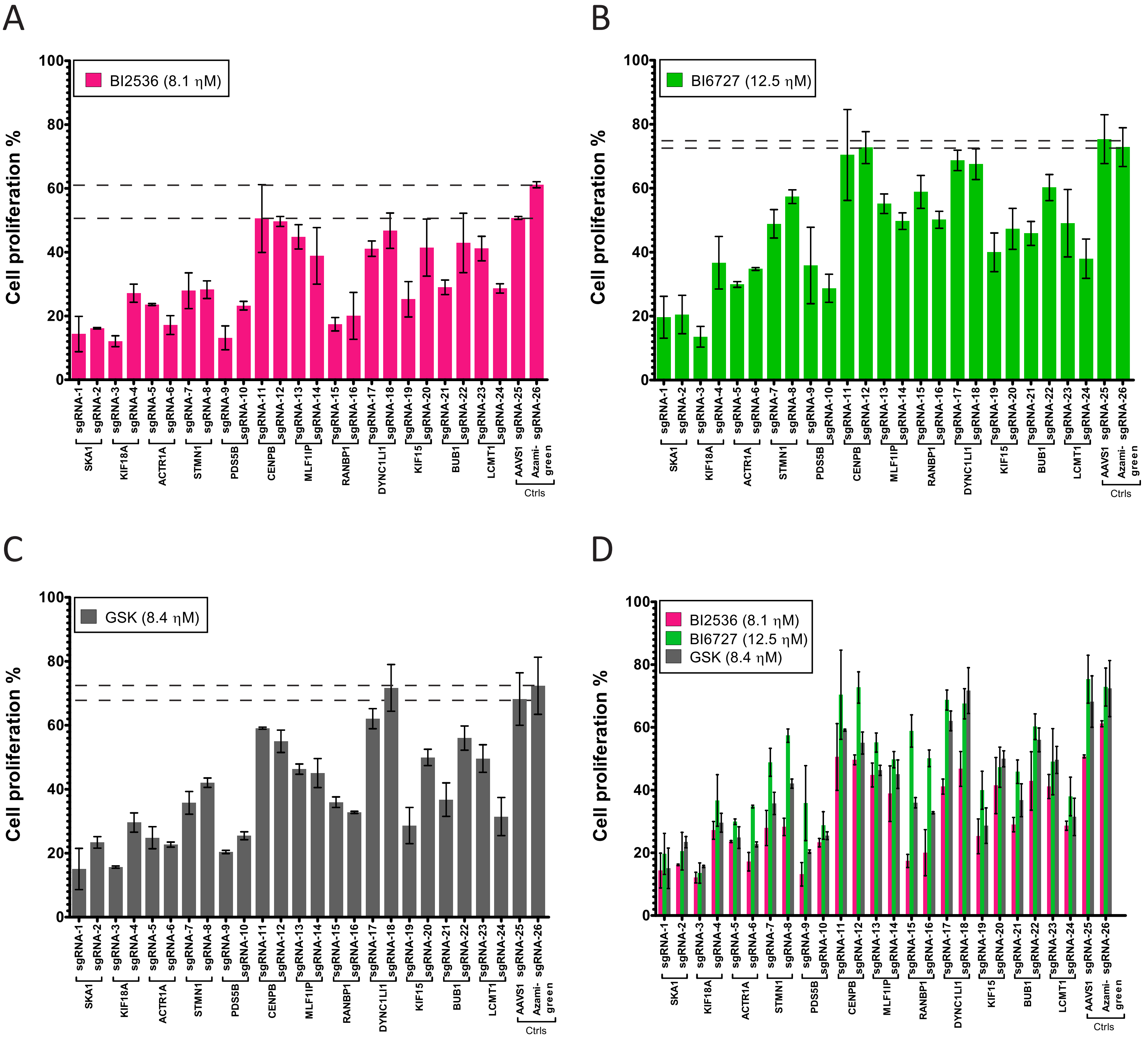

Figure S1. Validation of selected PLK1 inhibition enhancers. NALM-6 cells were infected with two different sgRNA constructs per gene and with 2 control sgRNAs. After selection with antibiotics, cells were treated with the indicated drugs at the IC_{30} concentrations or with DMSO for 96 hours. Percentages of cell proliferation in the presence of the drug relative to the DMSO control for each cell line are shown. Error bars: range of values from 2 independent experiments. A. BI2536 8.1 nM. B. BI6727 12.5 nM.C. GSK461364A 8.4 nM. D. All results in parallel, showing correlation. Dashed lines: % proliferation of the 2 controls. Coordinate values used to generate graphs are available in supplemental file Fig S1 Numerical Data.
