# Supplementary material for: Genetic enhancers of partial PLK1 inhibition reveal hypersensitivity to kinetochore perturbations
Source: PLoS Genet. 2023 Aug 28;19(8):e1010903. doi: 10.1371/journal.pgen.1010903 (PMC10491399; doi:10.1371/journal.pgen.1010903)
Supplement: S2 Fig — A. Example images of phenotypes observed by immunofluorescence in RPE-1 cells depleted of PRR14L using siRNA and treated with 5 nM BI2536 (IC30). B. Validation of PRR14L siRNA efficiency. C. Quantification of binucleated and multinucleated cells observed after the indicated treatments. Values are averages of 4 experiments in which >350 cells were scored per conditions in each experiment. ** p < 0.01 in Student unpaired T test. ns: non-significant. Coordinate values used to generate graphs are available in S9 Data. (PDF) [file pgen.1010903.s002.pdf]

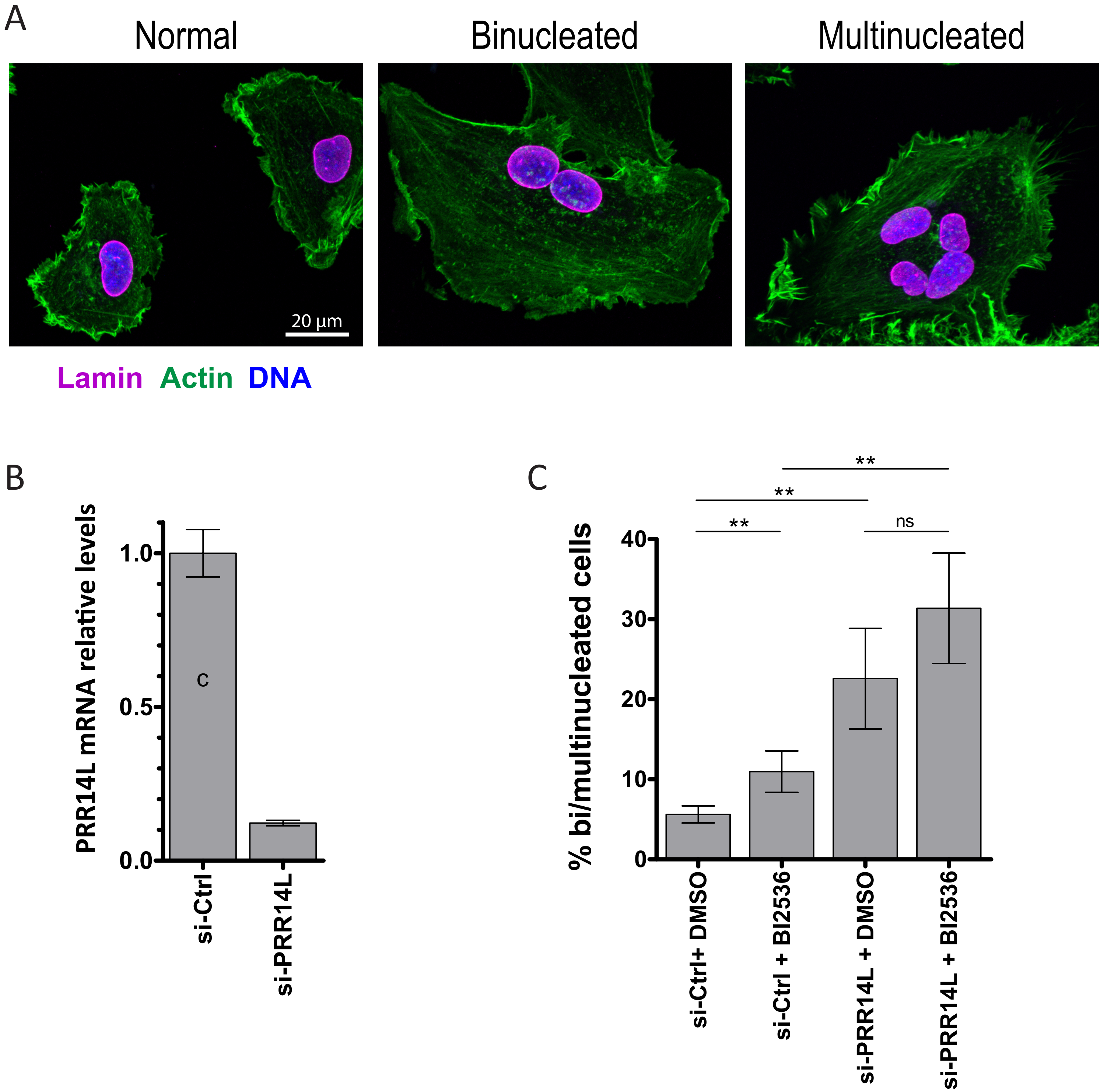

Figure S2. PRR14L is an enhancer of PLK1 inhibition in cytokinesis. A. Example images of phenotypes observed by immunofluorescence in RPE-1 cells depleted of PRR14L using siRNA and treated with 5 nM BI2536 (IC_{30}). B. Validation of PRR14L siRNA efficiency. C. Quantification of binucleated and multinucleated cells observed after the indicated treatments. Values are averages of 4 experiments in which >350 cells were scored per conditions in each experiment. ** $p < 0.01$ in Student unpaired T test. ns: non-significant. Coordinate values used to generate graphs are available in supplemental file Fig S2 Numerical Data.
