# Supplementary material for: Genetic enhancers of partial PLK1 inhibition reveal hypersensitivity to kinetochore perturbations
Source: PLoS Genet. 2023 Aug 28;19(8):e1010903. doi: 10.1371/journal.pgen.1010903 (PMC10491399; doi:10.1371/journal.pgen.1010903)
Supplement: S3 Fig — A. Positions targeted by the sgRNAs relative to known domains and motifs in the primary structure of KIF18A and SKA1. B. Indel distributions of NALM-6 cells selected for the expression of the indicated sgRNAs along with Cas9. C. Summary of data from NALM-6 CRISPR cell lines analyzed in B. Total % R2: percentage of the allelic population that could be reliably analyzed. D. Western blot analysis for KIF18A expression in the indicated cell lines. Coordinate values used to generate graphs are available in S10 Data. (PDF) [file pgen.1010903.s003.pdf]

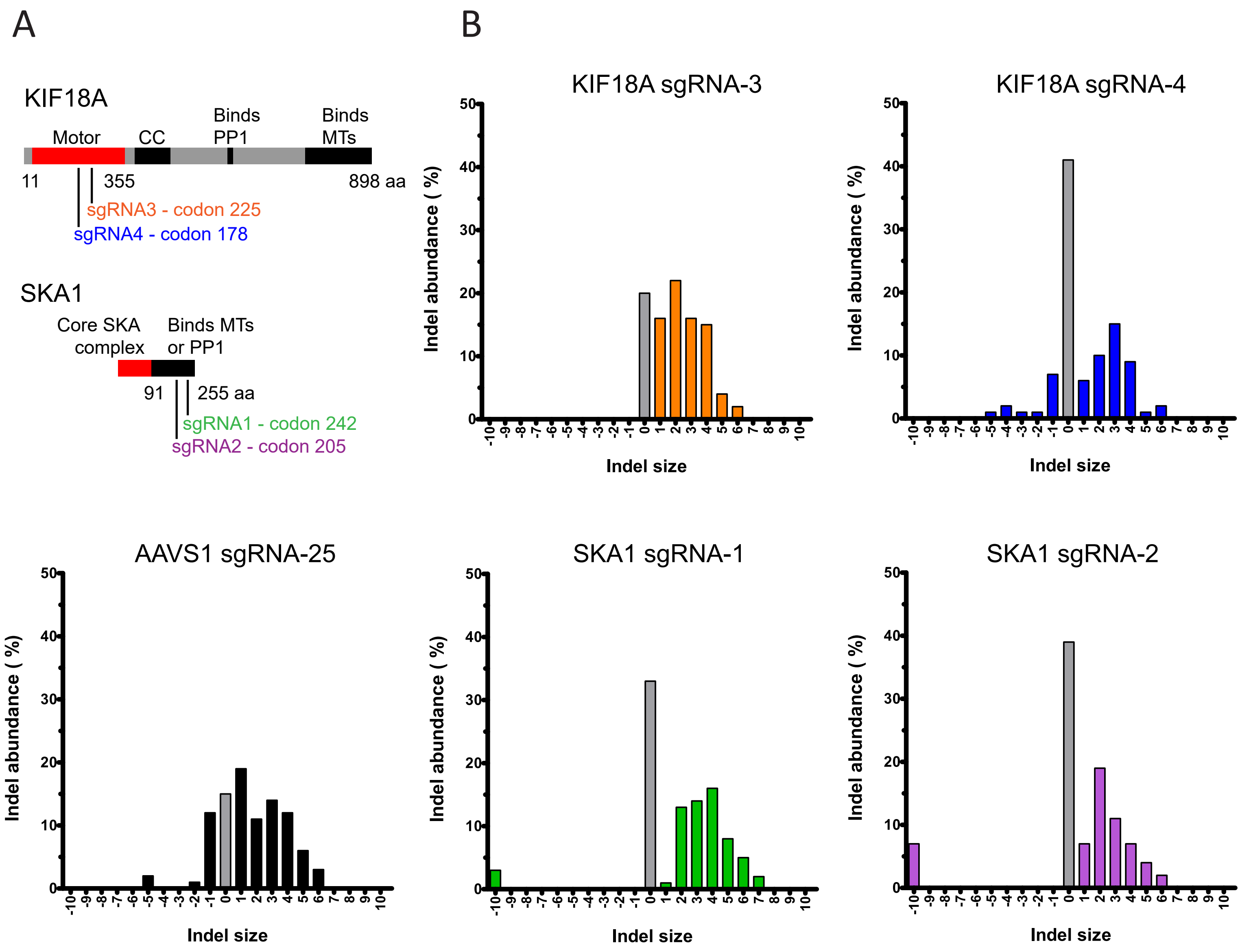

C

Name	Total % R^2	Indel %	WT %	Frame-shift %
**AAVS1 sgRNA-25**	95	80	15	63
**SKA1 sgRNA-1**	95	62	33	43
**SKA1 sgRNA-2**	96	57	39	44
**KIF18A sgRNA-3**	95	75	20	57
**KIF18A sgRNA-4**	96	55	41	37

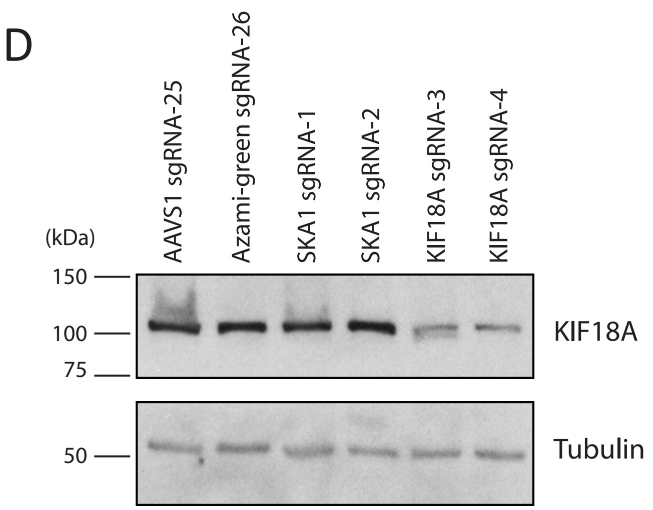

Figure S3. Analysis of NALM-6 cell lines obtained after CRISPR targeting of KIF18A and SKA1. A. Positions targeted by the sgRNAs relative to known domains and motifs in the primary structure of KIF18A and SKA1. B. Indel distributions of NALM-6 cells selected for the expression of the indicated sgRNAs along with Cas9. C. Summary of data from NALM-6 CRISPR cell lines analyzed in B. Total % R2: percentage of the allelic population that could be reliably analyzed. D. Western blot analysis for KIF18A expression in the indicated cell lines. Coordinate values used to generate graphs are available in supplemental file Fig S3 Numerical Data.
