# Supplementary material for: Genetic enhancers of partial PLK1 inhibition reveal hypersensitivity to kinetochore perturbations
Source: PLoS Genet. 2023 Aug 28;19(8):e1010903. doi: 10.1371/journal.pgen.1010903 (PMC10491399; doi:10.1371/journal.pgen.1010903)
Supplement: S4 Fig — A. Targeting KIF18A or SKA1 by CRISPR sensitizes NALM-6 cells to the GSK461364A-induced mitotic arrest. For A and B, the mitotic index was measured after immunofluorescence for pHH3. Representative experiments are shown. Data points are averages of triplicates ±SD. B. Silencing KIF18A or SKA1 by siRNA sensitizes hTERT RPE-1 cells to the GSK461364A-induced mitotic arrest. Coordinate values used to generate graphs are available in S11 Data. (PDF) [file pgen.1010903.s004.pdf]

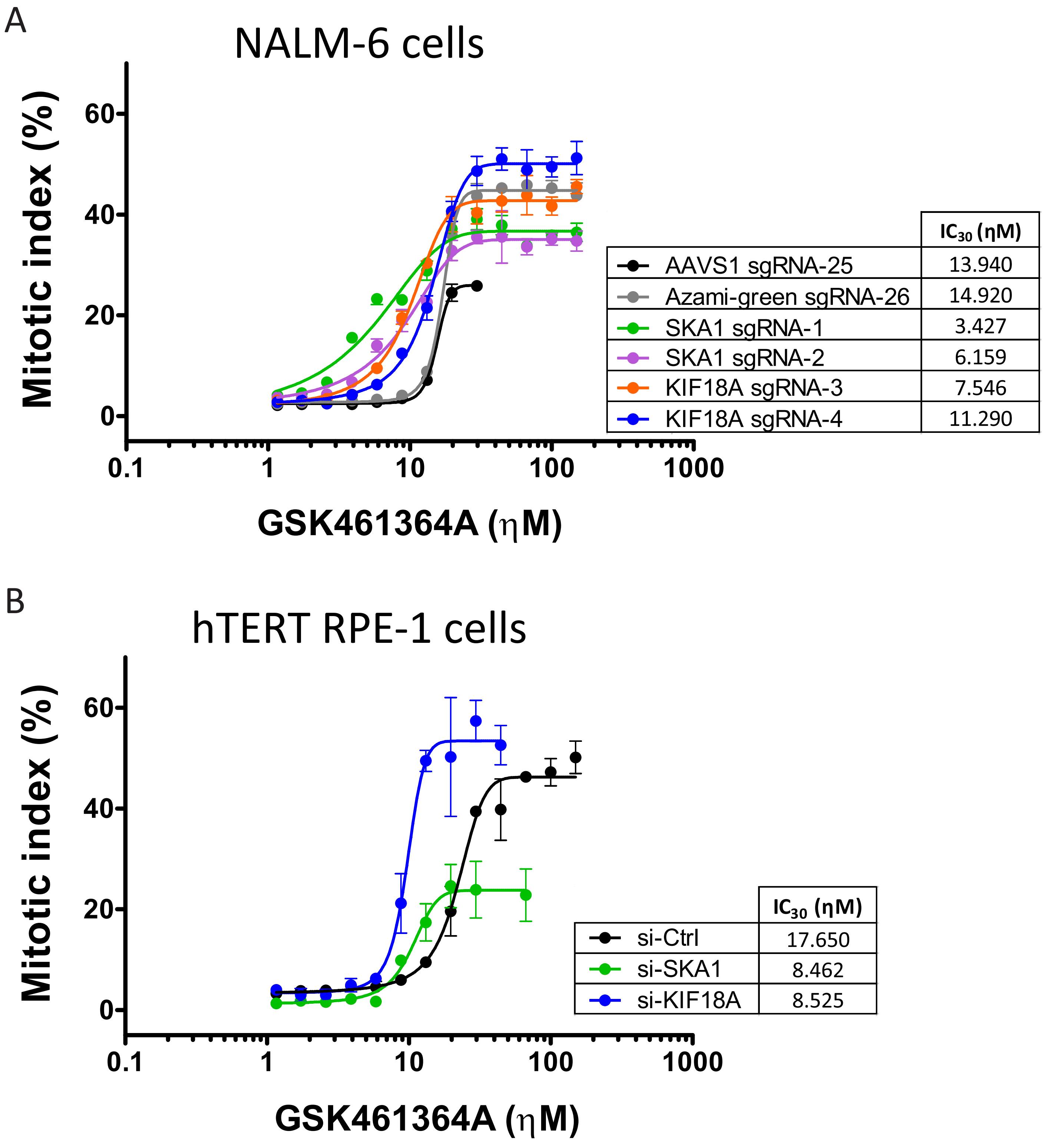

Figure S4. Inactivation of KIF18A or SKA1 sensitizes cells to mitotic arrest induced by PLK1 inhibition using GSK461364A. A. Targeting KIF18A or SKA1 by CRISPR sensitizes NALM-6 cells to the GSK461364A-induced mitotic arrest. For A and B, the mitotic index was measured after immunofluorescence for pHH3. Representative experiments are shown. Data points are averages of triplicates ±SD. B. Silencing KIF18A or SKA1 by siRNA sensitizes hTERT RPE-1 cells to the GSK461364A-induced mitotic arrest. Coordinate values used to generate underlying graphs are available in supplemental file Fig S4 Numerical Data.
