# Supplementary material for: Genetic enhancers of partial PLK1 inhibition reveal hypersensitivity to kinetochore perturbations
Source: PLoS Genet. 2023 Aug 28;19(8):e1010903. doi: 10.1371/journal.pgen.1010903 (PMC10491399; doi:10.1371/journal.pgen.1010903)
Supplement: S5 Fig — A. Relative levels of SKA1 and KIF18A mRNA were quantified by RT-qPCR. Two independent experiments of siRNA depletions were done in duplicates, resulting in 4 values. Averages ±SD are shown. For each gene (SKA1 or KIF18A), values were normalized by setting the si-Ctrl + DMSO average to 1 (C: calibrator). BI2536 was added at the IC30 concentration (5 nM). B. Western blots showing the levels of KIF18A protein in all transfections. Coordinate values used to generate graphs are available in S12 Data. (PDF) [file pgen.1010903.s005.pdf]

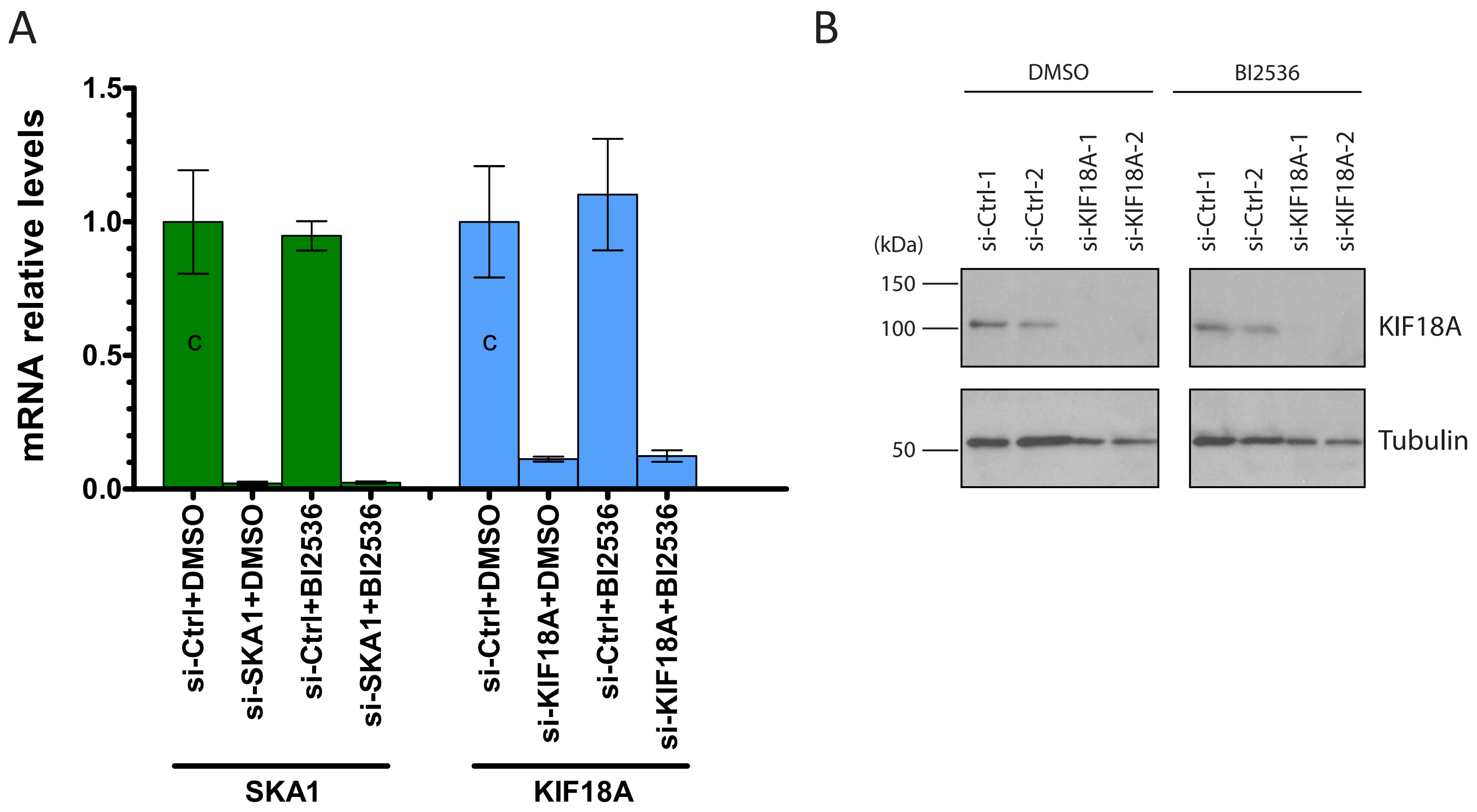

Figure S5. Validation of siRNA depletions in RPE-1 cells. A. Relative levels of SKA1 and KIF18A mRNA were quantified by RT-qPCR. Two independent experiments of siRNA depletions were done in duplicates, resulting in 4 values. Averages ±SD are shown. For each gene (SKA1 or KIF18A), values were normalized by setting the si-Ctrl + DMSO average to 1 (C: calibrator). BI2536 was added at the IC_{30} concentration (5 nM). B. Western blots showing the levels of KIF18A protein in all transfections. Coordinate values used to generate graphs are available in supplemental file Fig S5 Numerical Data.
