# Supplementary material for: Genetic enhancers of partial PLK1 inhibition reveal hypersensitivity to kinetochore perturbations
Source: PLoS Genet. 2023 Aug 28;19(8):e1010903. doi: 10.1371/journal.pgen.1010903 (PMC10491399; doi:10.1371/journal.pgen.1010903)
Supplement: S3 Table — Oligos used in RT-qPCR to measure the mRNA levels of the indicated genes. Reference of the detected isoform(s) according to NCBI, Universal Probe Library number used and the efficiency of each pair of oligos following a standard curve for each gene tested are included (see Materials & Methods). (PDF) [file pgen.1010903.s008.pdf]

Gene	UPL Probe	Oligo FWD	Oligo REV	RefSeq of isoform detected	Efficiency (%)
SKA1	30	aacctcccaggctcaagtg	gcttgtagtcccagccattc	NM_001039535.2 NM_145060.3	103
KIF18A	56	actgcagtttcatatctggaggt	ccacccctttttgggtatct	NM_031217.3	103
PRR14L	61	aggtagacctggcgacgac	tcaagtcttttacatcaaatgattcac	NM_173566.2	100
ACTB	11	attggcaatgagcggttc	tgaaggtagtttcgtggatgc	NM_001101.3	97
HPRT	22	tgatagatccattcctatgactgtaga	caagacattctttccagttaaagttg	NM_000194.2	99

Table S3. Oligonucleotides used in RT-qPCR in this study. Oligos used in RT-qPCR to measure the mRNA levels of the indicated genes. Reference of the detected isoform(s) according to NCBI, Universal Probe Library number used and the efficiency of each pair of oligos following a standard curve for each gene tested are included (see Materials & Methods).
