# Supplementary material for: Genetic enhancers of partial PLK1 inhibition reveal hypersensitivity to kinetochore perturbations
Source: PLoS Genet. 2023 Aug 28;19(8):e1010903. doi: 10.1371/journal.pgen.1010903 (PMC10491399; doi:10.1371/journal.pgen.1010903)
Supplement: Attachment — Submitted filename: Response to Reviewers Normandin et al.pdf [file pgen.1010903.s029.pdf]

Dear Drs Snyder and Kwiatkowski,

We were pleased by your decision that our manuscript will be acceptable for publication after minor revisions. We thank the reviewers for their thoughtful comments and suggestions. Please find below our detailed responses, in blue.

Reviewer #1: In this manuscript, the authors report results from a genome-wide CRISPR screen designed to identify enhancers and suppressors of PLK1 inhibitor-dependent suppression of cell proliferation. The screen utilized three different PLK1 inhibitors and identified a number of modifiers involved in cell cycle regulation, kinetochore assembly and function, and the microtubule cytoskeleton. A number of the strongest enhancers of PLK1 have known roles in kinetochore function or regulation of kinetochore microtubule dynamics, suggesting that the kinetochore functions of PLK1 may be particularly dependent on high PLK1 activity. The authors chose two of the identified enhancers, SKA1 and KIF18A, to investigate further in NALM-6 and RPE-1 cells. Combining PLK1 inhibitors at IC30 with siRNAs targeting SKA1 or KIF18A resulted in severe chromosome alignment and mitotic arrest defects, as well as nuclear shape abnormalities. While the mechanistic basis of the enhancer effects from SKA1 and KIF18A knockdown were not completely addressed, the data presented are convincing and provide an important step towards identifying combinatorial, anti-mitotic treatments worthy of additional exploration. A few minor concerns are provided below for the authors to consider.

1. Since PLK1 has a known role in CENP-A recruitment, it could be informative to determine if the severity of mitotic phenotypes scales with CENP-A levels at centromeres.

We thank the reviewer for this key suggestion. We note that it would be technically difficult to make reliable observations on a single-cell basis given the variability of immunofluorescence signals combined with the highly dynamic development of the phenotypes following treatments. Nevertheless, we agree that the role of PLK1 in CENP-A recruitment is a likely mechanistic reason underlying the phenotypes we observed. We reasoned that if this is the case, then CENP-A loading should be highly sensitive to partial inhibition of PLK1. To test this idea, we have measured how centromeric CENP-A levels change after treating RPE-1 cells with different concentrations of PLK1 inhibitor (BI2536). Consistent with this model, we found that low concentrations of BI2536 (even at the IC_{10} for proliferation) cause a marked reduction in CENP-A levels. These new data are presented in the new Figure 7 and accompanying text. These results strengthen the mechanistic insights of our study by suggesting that CENP-A loading may be the essential function of PLK1 that is most sensitive to its partial inhibition.

2. In Figure 5, Ska1 siRNA cells appear to be multipolar. Were these included in the centromere distribution analyses? If so, it should be explained how these

results were compared to those from bipolar spindles and how they might affect the dispersion ratios presented.

We did in fact observe many multipolar spindles upon SKA1 depletion. Multipolar spindles were included in the quantification, where we measured centromere dispersion along the longest axis of the spindle. We have added the following sentence to make this point explicit:

"SKA1 depletion increased the occurrence of multipolar spindles, which could contribute to increase centromere dispersion, as quantified along the longest axis of the spindle."

3. The authors may want to consider avoiding the term "nuclear reassembly defects" to describe the nuclear shape abnormalities reported in Figure 6. "Nuclear reassembly" suggests the process of nuclear envelope formation after mitosis. It is not clear from the data presented that the integrity of the nuclear envelope was tested, and I think the authors are likely referring to defects in forming a single nucleus around all chromosomes.

We indeed refer to defects informing a single nucleus with a normal shape, and not merely to the process of nuclear envelope reformation. We have now defined the term at its first use in the Results section.

Reviewer #2: In the manuscript by Normandin, et. al., the authors probe the genome for proteins whose depletion is synthetically interactive with Polo Like Kinase 1(PLK1) inhibition. They identify numerous interactors involved in the cell cycle, unsurprisingly. Further evaluation leads the authors to conclude that combination therapies between PLK1 and proteins involved in chromosome segregation, mitotic spindle function, and nuclear reformation in G1, among others, would be a promising strategy for clinical exploration.

PLK1 is an important cell cycle kinase that has been targeted for cancer therapeutics. PLK1 has long been known to regulate numerous mitotic events including but not limited to centrosome maturation, chromosome condensation, kinetochore function, cytokinesis, and various aspects of mitotic exit including centromere epigenetic regulation. Possibly due to these pleiotropies, PLK1 inhibitors have seen limited success in drug discovery. Here the authors identify the mitotic kinesin KLP18a, a plus end directed family 8 kinesin, as a potential target for combinatorial therapies.

Overall this is a well conducted study that probes a possibly critical question in therapeutics, identification of cocktail type treatments that result in better targeting of cancerous cell types. I am generally in favor of accepting this paper, however I have some reservations that dampen my enthusiasm.

1) The main concern I have is the lack of new insight into mitotic mechanisms. I left this paper feeling like I could have predicted every interactor discovered based on previous publications. This is likely not the main point of the paper, however still wish I had learned more about mitosis from the work. In looking at the figures (5A for instance) kinetochores appear to have lost "integrity" as discrete loci. I find this very interesting, among other phenotypes, and hope the authors follow on with further, more basic and mechanistic studies in the future.

This is an interesting observation and we thank the reviewer for pointing it out. We plan to investigate the precise mechanistic reason for this aspect of the phenotype in the future.

Regarding the limited mechanistic insight of our study, please note that we have now conducted new experiments as described in response to the first point of Reviewer 1.

2) There have been few cell level retrospectives on how cancer drugs such as Taxol/Paclitaxol kill cells. Mitchison has conducted a series of studies on Taxol treatment at clinically relevant doses and found that, if I may oversimplify, catastrophic exit from mitosis is the predominant form of death. This seems similar to the observation here of nuclear reformation etc. It might be nice to have some discussion of this point.

We thank the reviewer for this thought and we have now made this interesting connection in the discussion (last paragraph).

3) Along those lines, I find the most important part of this is not the cell biology (which is not so insightful), rather the clinical implications. More of an observation than anything else.

We agree, and we have further emphasized the potential clinical relevance of our findings in the discussion.

4) Finally, the centromere dispersion assay is not very informative in this context. Many spindle/kinetochore perturbations produce essentially the same thing, increased dispersion of centromeres on the spindle. Live cell imaging to measure congression would be a more appropriate assay.

We have indeed used live cell imaging and observed congression defects in cells depleted of KIF18A or SKA1, in addition to the mitotic arrests prior to anaphase (Fig 4). However, we do not think it is essential to quantify the rate of congression to support our conclusions. We only quantified centromere dispersion in metaphase because we wanted to assess if defects at that level could underly subsequent anaphase delays or defects we observed. Since we observed no significant enhancement of centromere dispersion when PLK1 was inhibited in addition to KIF18A or SKA1 depletion, we reason that defects in congression are

unlikely to be the underlying cause of the enhanced mitotic arrests and nuclear reassembly defects. Instead, weaker kinetochore attachments may enhance defects specifically in anaphase. We have added this point in the discussion.

Thank you very much for your help and please let us know if you need anything else from us.

Regards,

Vincent Archambault
